# Small RNA sequencing analysis of peptide-affinity isolated plasma extracellular vesicles distinguishes pancreatic cancer patients from non-affected individuals

**DOI:** 10.1038/s41598-023-36370-3

**Published:** 2023-06-07

**Authors:** Jeremy W. Roy, Gabriel Wajnberg, Alexie Ouellette, Julie Emilie Boucher, Jacynthe Lacroix, Simi Chacko, Anirban Ghosh, Rodney J. Ouellette, Stephen M. Lewis

**Affiliations:** 1 Atlantic Cancer Research Institute, Moncton, NB Canada; 2 Beatrice Hunter Cancer Research Institute, Halifax, NS Canada; 3 Department of Chemistry and Biochemistry, Université de Moncton, Moncton, NB Canada; 4 Dr. Georges-L.-Dumont University Hospital Centre, Moncton, NB Canada

**Keywords:** Tumour biomarkers, Pancreatic cancer, Diagnostic markers

## Abstract

Pancreatic ductal adenocarcinoma (PDAC) has a high fatality rate, mainly due to its asymptomatic nature until late-stage disease and therefore delayed diagnosis that leads to a lack of timely treatment intervention. Consequently, there is a significant need for better methods to screen populations that are at high risk of developing PDAC. Such advances would result in earlier diagnosis, more treatment options, and ultimately better outcomes for patients. Several recent studies have applied the concept of liquid biopsy, which is the sampling of a biofluid (such as blood plasma) for the presence of disease biomarkers, to develop screening approaches for PDAC; several of these studies have focused on analysis of extracellular vesicles (EVs) and their cargoes. While these studies have identified many potential biomarkers for PDAC that are present within EVs, their application to clinical practice is hindered by the lack of a robust, reproducible method for EV isolation and analysis that is amenable to a clinical setting. Our previous research has shown that the Vn96 synthetic peptide is indeed a robust and reproducible method for EV isolation that has the potential to be used in a clinical setting. We have therefore chosen to investigate the utility of the Vn96 synthetic peptide for this isolation of EVs from human plasma and the subsequent detection of small RNA biomarkers of PDAC by Next-generation sequencing (NGS) analysis. We find that analysis of small RNA from Vn96-isolated EVs permits the discrimination of PDAC patients from non-affected individuals. Moreover, analyses of all small RNA species, miRNAs, and lncRNA fragments are most effective at segregating PDAC patients from non-affected individuals. Several of the identified small RNA biomarkers have been previously associated with and/or characterized in PDAC, indicating the validity of our findings, whereas other identified small RNA biomarkers may have novel roles in PDAC or cancer in general. Overall, our results provide a basis for a clinically-amendable detection and/or screening strategy for PDAC using a liquid biopsy approach that relies on Vn96-mediated isolation of EVs from plasma.

## Introduction

Pancreatic ductal adenocarcinoma (PDAC) has a very high mortality rate due to the significant number of patients (~ 80%) that have non-localized metastatic disease at the time of diagnosis^1^. Survival from PDAC is significantly higher the earlier the disease is diagnosed, at a time when complete surgical removal of the tumour offers the best chance at curative treatment^2^. Therefore, a key challenge for PDAC is the development of a reliable assay that will detect the disease at its earliest stages. Due to the asymptomatic nature of early-stage PDAC, populations that are at high-risk of developing this disease would derive significant benefit from preventative or diagnostic screening to identify its presence. Such populations include patients that have a family history of PDAC, suffer from chronic pancreatitis, or have type II diabetes^3^.

Liquid biopsy, which is the interrogation of biofluids (such as blood, urine, saliva, etc.) for the presence of disease biomarkers^4^, represents a promising approach for PDAC screening and detection. Typically, nucleic acids or proteins that circulate in a biofluid are analyzed during liquid biopsy; however, isolation of extracellular vesicles (EVs) from a biofluid and characterization of their cargoes, which include nucleic acids and proteins, is gaining interest^4^. Recent studies have explored the use of EV cargoes as biomarkers for PDAC^5–15^. While such studies have identified differential expression of nucleic acids and proteins in the EVs of PDAC patients, the robust application of these novel biomarkers in a clinical setting requires reliable techniques for the isolation of EVs in a high-purity homogeneous form, and these methods must be amenable for use in a clinical diagnostic laboratory^16^.

It has been well-established that the Vn96 synthetic peptide facilitates isolation of EVs from a variety of biofluids using a simple protocol that relies on standard laboratory equipment^17–24^. As such, the use of Vn96 enables multi-omic analysis of EV cargos including DNA, RNA and protein^23^. Small RNA sequencing of nucleic acid extracted from Vn96-captured EVs demonstrates an enrichment of EV small RNA and a similar sequencing profile to other commercially available EV isolation methods (ExoRNeasy), as well as the most commonly-used laboratory technique for EV capture (ultracentrifugation^23,25^). In a pre-clinical setting, Vn96 has been used to isolate and detect relevant disease biomarkers from plasma for amyotrophic lateral sclerosis^26^ and lung cancer^24^, and to isolate and detect biomarkers from urine for prostate cancer^18^. Together, these studies indicate that the Vn96 synthetic peptide is a promising candidate for an EV isolation method that is robust, reproducible, and applicable for a clinical diagnostics laboratory.

In the present study we used the Vn96 synthetic peptide to isolate EVs from plasma samples obtained from PDAC patients and non-affected individuals, followed by isolation of RNA and next-generation sequencing (NGS) analyses for small RNA species. We identified differentially-expressed small RNA species in EVs isolated from PDAC patients, with the most profound differences in the expression profiles of miRNAs and lncRNA fragments between PDAC patients and non-affected individuals. Moreover, our analyses identified small RNAs that were previously found to be associated with PDAC. Our results indicate that EVs isolated with the Vn96 synthetic peptide are able to discriminate between PDAC patients and non-affected individuals through analysis of their small RNA cargoes, providing a basis for a clinically-amendable detection and/or screening strategy for PDAC using a liquid biopsy approach.

## Results

### Small RNA sequencing analysis of Vn96-isolated EVs from the plasma of PDAC patients and non-affected individuals

We set out to study the differential expression of small RNAs, including miRNA, tRNA, and piRNA, found in EVs isolated with the Vn96 synthetic peptide from the plasma of patients with pancreatic ductal adenocarcinoma (PDAC) and non-affected ‘healthy’ control individuals. PDAC patients’ and non-affected individuals’ characteristics are shown in Table 1. There are no major differences for age and sex between the non-affected individuals and the PDAC patients. For our study we focused on PDAC patients who had disease stages of IIb and above with the goal of identifying a unifying small RNA profile of PDAC regardless of disease stage.

**Table 1:** Non-affected individuals’ and PDAC patients’ characteristics.

**Non-affected individuals**	
Age in years	
Mean	64
Median	66
Range	52–77
Sex	
Male (%)	7/13 (54%)
Female (%)	6/13 (46%)

**PDAC patients**	
Age in years	
Mean	70
Median	73
Range	50–85
Sex	
Male (%)	9/16 (56%)
Female (%)	7/16 (44%)
Disease stage	
> Stage IIb	16/16 (100%)

**Figure 1:**
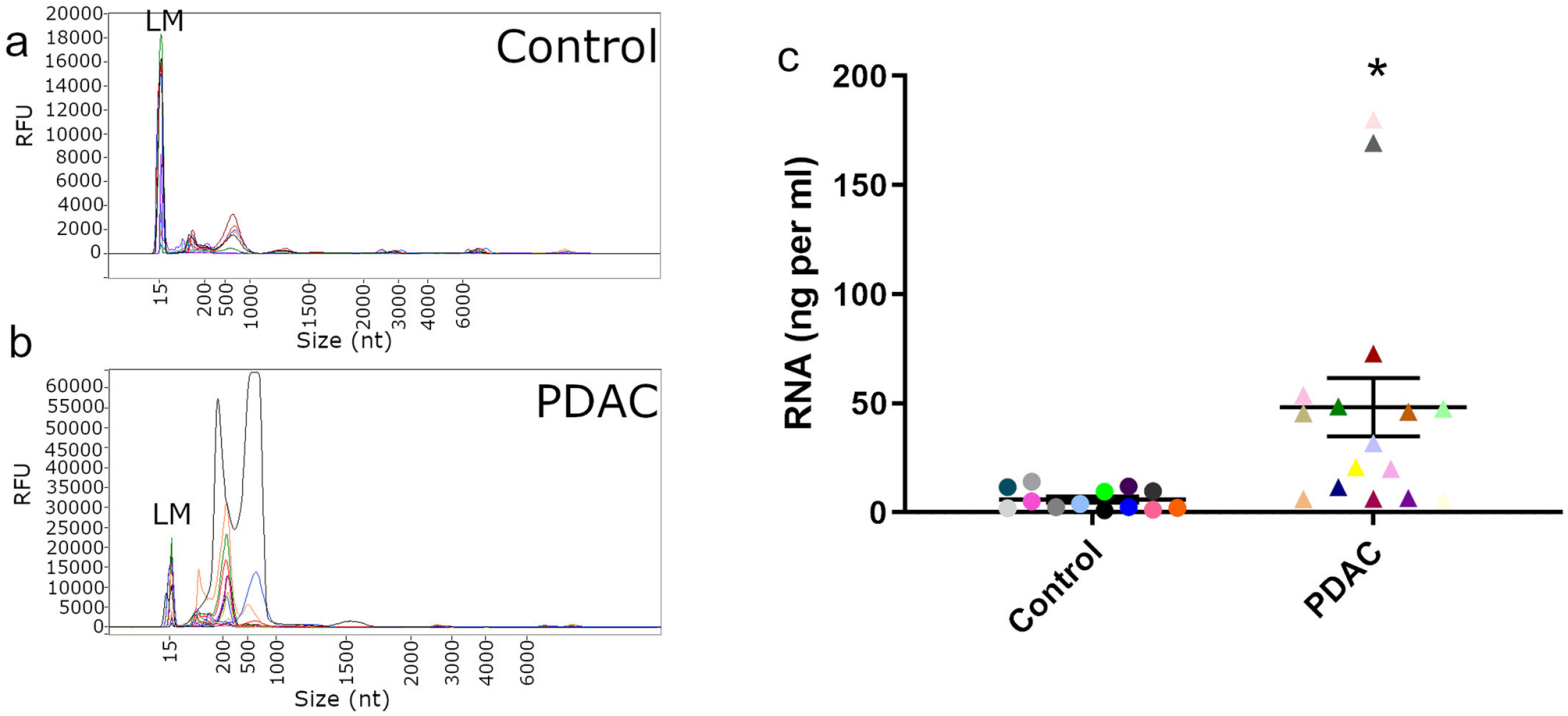
Fragment Analyzer results for Vn96-isolated EV-RNA. RNA extracted from EVs isolated from the plasma of (**a**) non-affected individuals (Control) and (**b**) PDAC patients was analyzed using the HS Total RNA kit. Note the absence of 18S and 28S rRNA and presence of small RNA typical of EV-RNA. Lower molecular weight size marker is indicated by LM. (**c**) Total Vn96-isolated EV-RNA yield (ng) per mL of plasma for non-affected individuals (Control) and PDAC patients (n = 29, *p = 0.0086).

In total, plasma samples from 13 non-affected individuals and 16 PDAC patients were subjected to EV isolation using a previously-established protocol that relies on the Vn96 synthetic peptide^22^. Total RNA was extracted from the isolated EVs and RNA profiles for non-affected individuals and PDAC patients are shown in Fig. 1. All samples have a typical EV RNA profile, which includes multiple peaks of small RNA below 1000 nucleotides and are devoid of 18S and 28S rRNA (Fig. 1a,b). Interestingly, the total amount of RNA (ng) extracted from EVs per mL of plasma is statistically different between non-affected individuals and PDAC patients (n = 29, P < 0.05; Fig. 1c). In two PDAC patients, the amount of EV RNA per mL of plasma is 3 times higher than the average for the PDAC patient group. These findings suggest either increased EV numbers in the plasma of PDAC patients or a significant enrichment of small RNA within the EVs of PDAC patients compared to non-affected individuals.

**Figure 2:**
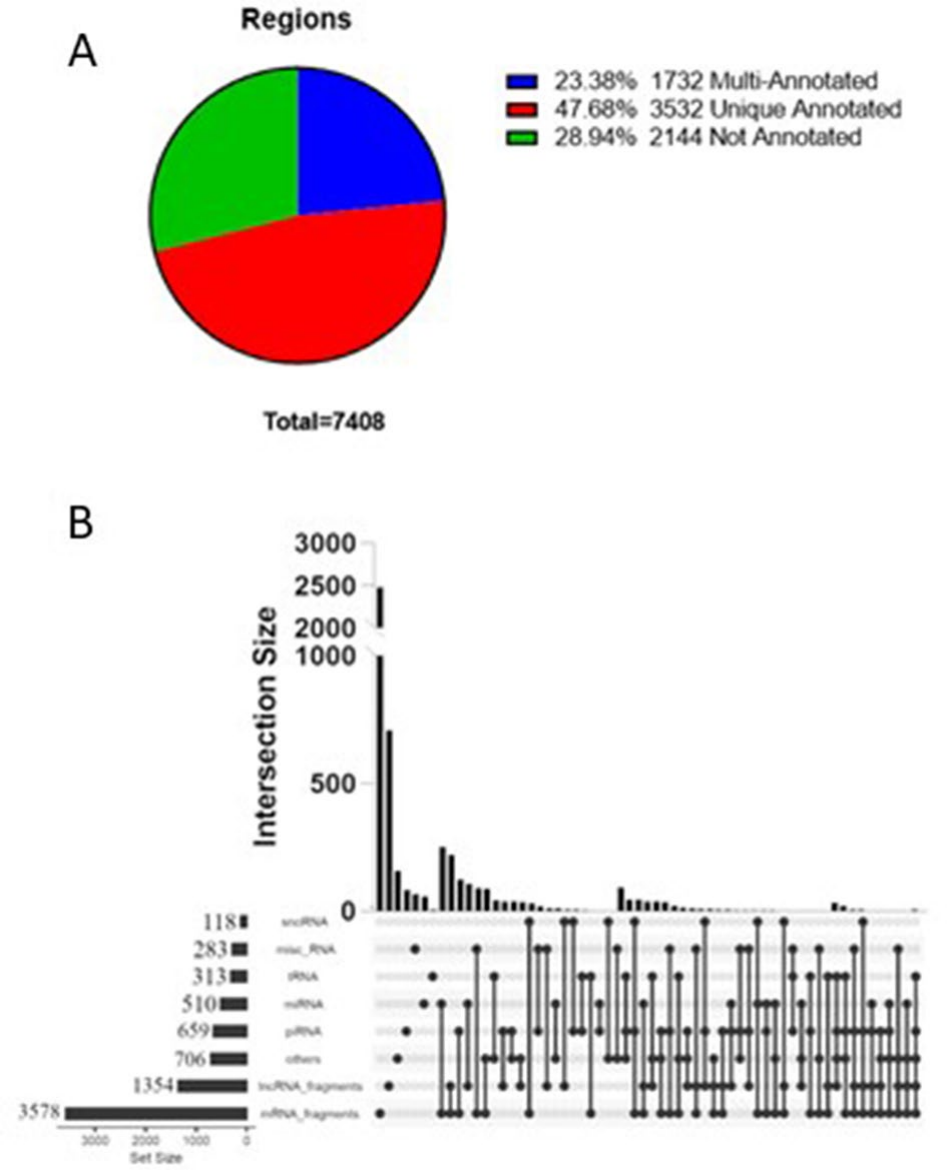
Genomic mapping and annotation results for small RNA sequencing of EV-RNA from non-affected individuals and PDAC patients (n = 29). **A**) Sequences were mapped to 7408 chromosomal regions, of which 3532 are uniquely annotated (47.68%), 1732 multi-annotated (23.38%) and 2144 not annotated (28.94%). **B**) Regions that are uniquely and multi-annotated include mRNA fragments, lncRNA fragments and small RNA species such as piRNA, miRNA, tRNA, misc_RNA, snoRNA and others.

**Figure 3:**
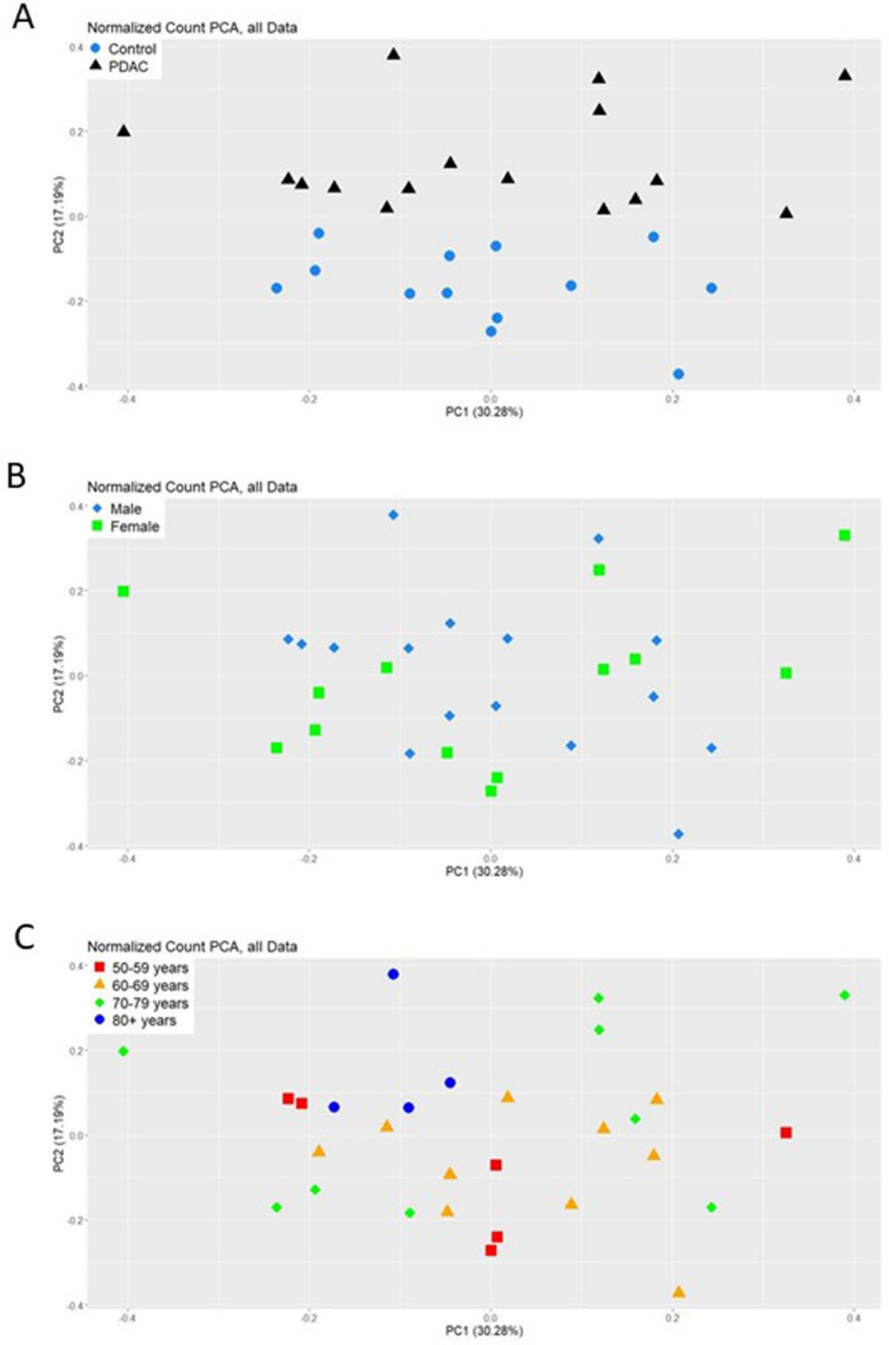
Multidimensional scaling (MDS) scatterplots of all 7408 chromosomal regions. (**A**) Points labelled with condition, non-affected individuals (Control, blue circles) and PDAC patients (black triangles). (**B**) Points labelled with sex, male (blue diamonds) and female (green squares). (**C**) Points labelled with age separated by decades, 50–59 years (red squares), 60–69 years (orange triangles), 70–79 years (green diamonds) and 80+ years (blue circles).

We performed a Next-Generation Sequencing (NGS) analysis for small RNAs (sRNAseq) on all EV-RNA samples as described in the Methods section. In the present study we chose to use the R package derfinder^27^, which aligns sequencing data to the genome to first provide chromosomal regions, then annotates these regions using available databases. This type of approach has previously been used for RNA sequencing data derived from cellular RNA^28,29^, and our validation of this methodology for the analysis of small RNAs from EVs is described in detail elsewhere^30^. Analysis of our sRNAseq data determined that there are no statistically-significant differences for total reads obtained and reads passing filters (Supplemental Fig. 1a and b) between PDAC patients and non-affected individuals, whereas samples from PDAC patients have statistically-significant increases in reads mapped , percentage of reads mapped, and average read length compared to non-affected individuals (Supplemental Fig. 1C, D, and E). The increase in read mapping for PDAC patients may reflect a higher complexity of small RNA cargoes in the EVs from this population. In the total dataset (n = 29), we identified 7408 chromosomal regions that passed filters, as described in the Methods section, in the RNA isolated from PDAC patients’ EVs and EVs from non-affected individuals, of which 47.68% are uniquely annotated, 28.94% are not annotated (NA), and 23.38% are multi-annotated (Fig. 2A). Of the annotated regions (5264 in total), 67.97% are annotated to mRNA fragments, 25.72% are annotated to lncRNA fragments, 12.55% are annotated to ‘other’ RNA, 12.52% are annotated to piRNA, 5.56% are annotated to miRNA, 5.38% are annotated to tRNA, 2.24% are annotated to misc_RNA, and 1.59% are annotated to snoRNA (Fig. 2B). We then subjected our sRNAseq dataset (all 7408 chromosomal regions) to multidimensional scaling (MDS) scatterplot analysis. MDS for condition (PDAC versus non-affected) shows good segregation of samples (Fig. 3A), whereas MDS for sex (Fig. 3B) and age (Fig. 3C) demonstrate that there is no significant segregation of samples based on these characteristics. These results indicate that disease condition is the most significant difference that contributes to the segregation of PDAC patients’ and non-affected individuals’ EV small RNA profiles that were generated by sRNAseq analysis of small RNA extracted from Vn96-isolated EVs.

**Figure 4:**
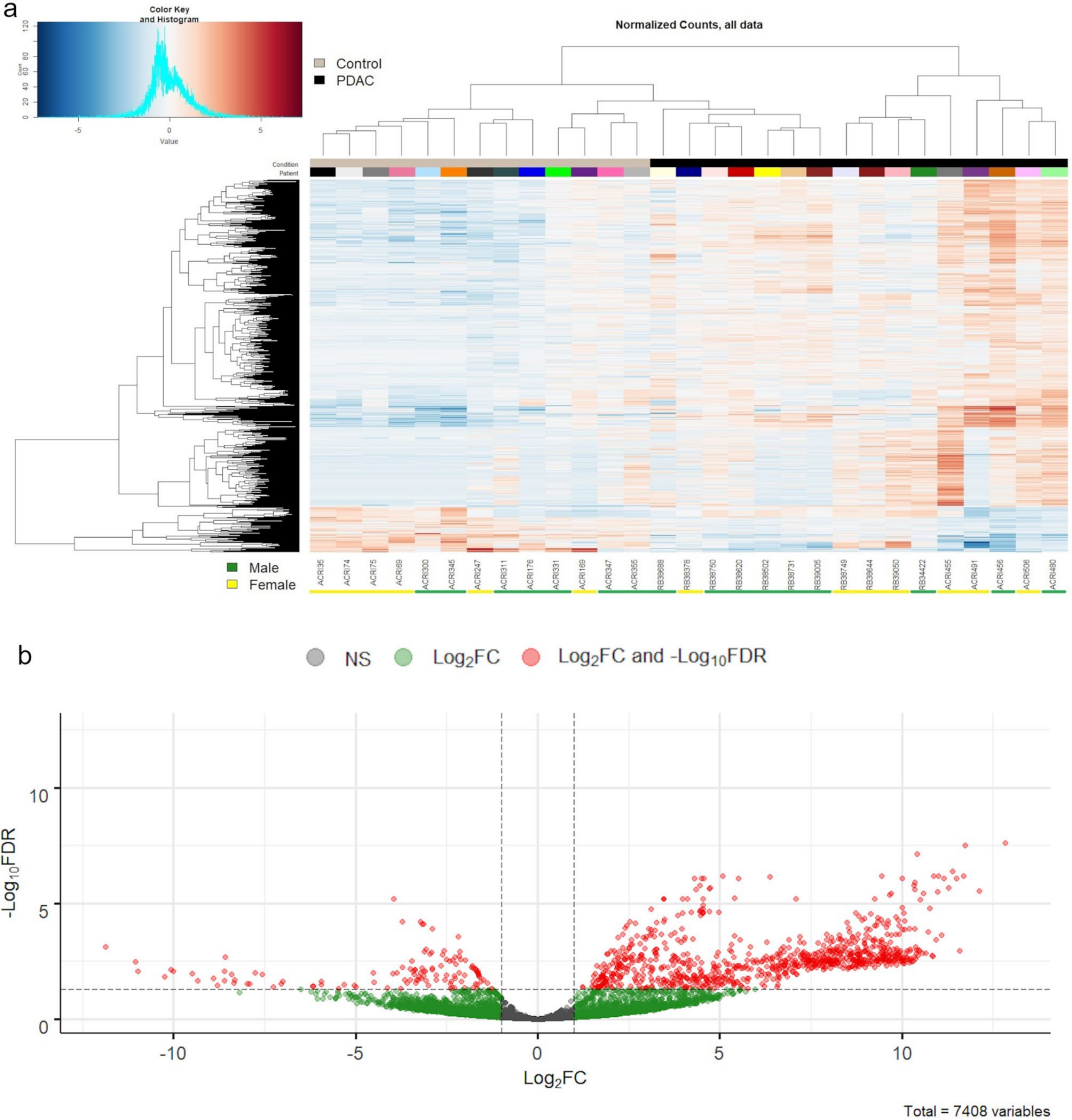
Analysis of all 7408 chromosomal regions for non-affected individuals (Control; grey) and PDAC patients (black). (**a**) The heatmap represents log transformed normalized (TMM) reads. Heatmap colors correspond to RNA expression as indicated in the color key: blue (down-regulated) and red (up-regulated). (**b**) Volcano plot demonstrating the results of differential analysis between non-affected individuals (n = 13) versus PDAC patients (n = 16). Statistically significant chromosomal regions that are both FDR < 0.05 and Log_2_FC >  ± 1.0 are highlighted in red. Statistically significant chromosomal regions that are Log_2_FC >  ± 1.0 but not FDR < 0.05 are highlighted in green. FDR = false-discovery rate; negative Log_2_ fold-change (Log_2_FC) values represent decreased expression in PDAC patients’ samples; positive Log_2_ fold-change (Log_2_FC) values represent increased expression in PDAC patients’ samples; NS = not significant.

We then performed an unsupervised hierarchal clustering analysis using all 7408 regions, which generated the hierarchical clustering and heat map shown in Fig. 4a. This analysis showed all 7408 regions permit clustering of the majority of non-affected individuals, as well as clustering of the majority of PDAC patients. At the first branch point, all non-affected individuals and 7 of 16 PDAC patients cluster together, which are separated from 9 of 16 PDAC patients. Following the left side of the dendrogram, at the next branch point 9 of 13 non-affected individuals cluster together and are separated from the 7 of 16 PDAC patients and 4 of the non-affected individuals. Finally, these 7 of 16 PDAC patients cluster together and separate from the 4 non-affected individuals. Of note is the observation that there is a clear delineation between PDAC patients and non-affected individuals, as no PDAC or non-affected individuals cluster closely with one another and are not observed in the opposite group, strongly suggesting significant differences between the groups. Differential expression analysis of PDAC patients versus non-affected individuals is shown in the Volcano plot (Fig. 4b). We detected 875 up-regulated regions and 120 down-regulated regions in PDAC patients that are statistically significant (FDR < 0.05, log_2_FC >  ± 1.0). Overall, sRNAseq analysis of small RNA extracted from Vn96-isolated EVs permits the separation of PDAC patients from non-affected individuals, with each group having a unique EV small RNA expression profile.

### Analysis of the differential expression of specific small RNA species in Vn96-isolated EVs from PDAC patients and non-affected individuals

We next performed unsupervised hierarchal clustering analyses using each individual annotation category for the analysis, including miRNA, piRNA, tRNA, mRNA (fragments), and lncRNA (fragments). These analyses provide an understanding of the utility of each small RNA species for segregating PDAC patients from non-affected individuals.

**Figure 5:**
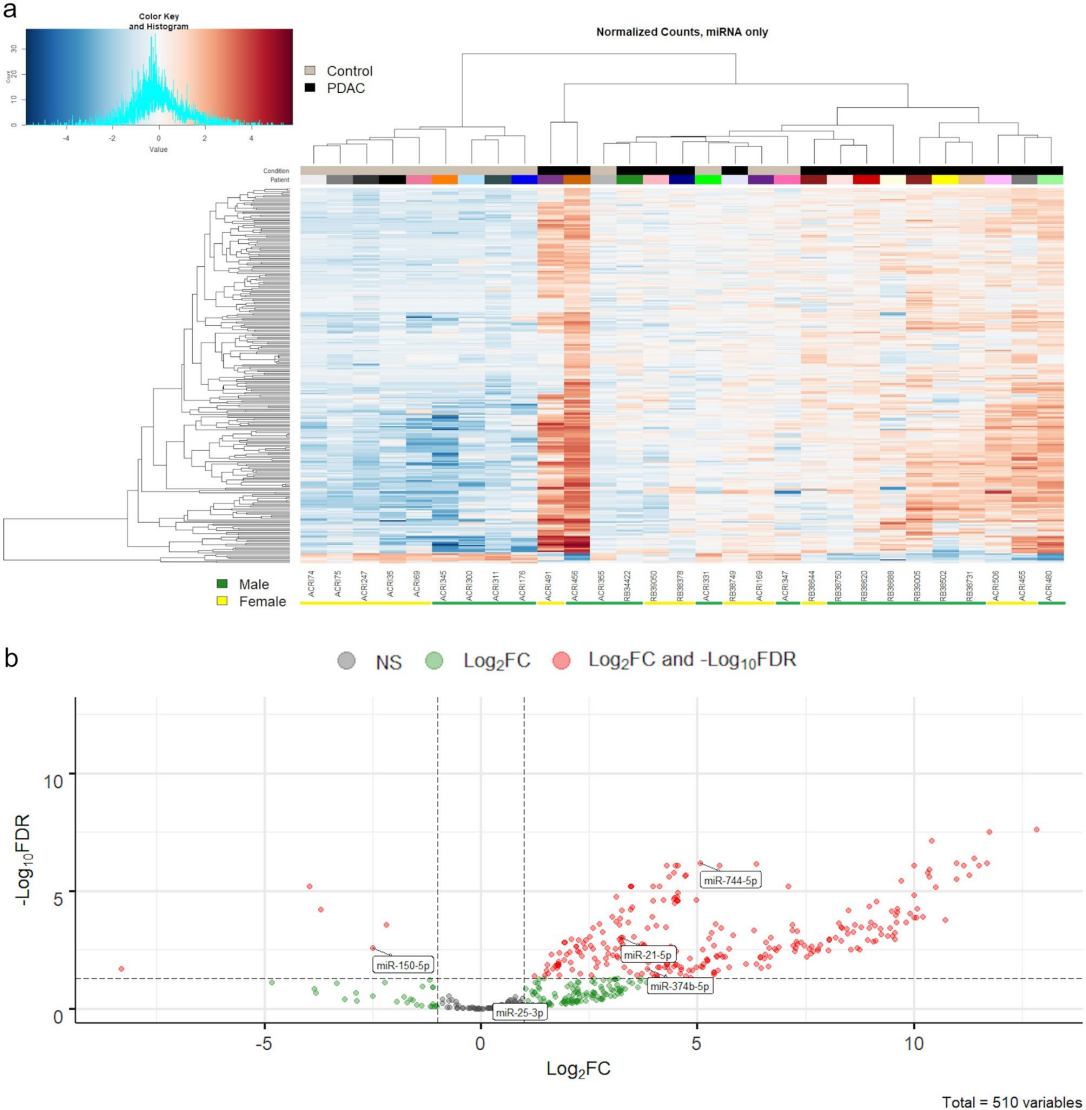
Analysis of annotated miRNA (510) for non-affected individuals (Control; grey) and PDAC patients (black). (**a**) The heatmap represents log transformed normalized (TMM) reads. Heatmap colors correspond to RNA expression as indicated in the color key: blue (down-regulated) and red (up-regulated). (**b**) Volcano plot demonstrating the results of differential analysis between non-affected individuals (n = 13) versus PDAC patients (n = 16). Statistically significant miRNA that are both FDR < 0.05 and Log_2_FC >  ± 1.0 are highlighted in red. Statistically significant miRNA that are Log_2_FC >  ± 1.0 but not FDR < 0.05 are highlighted in green. FDR = false-discovery rate; negative Log_2_ fold-change (Log_2_FC) values represent decreased expression in PDAC patients’ samples; positive Log_2_ fold-change (Log_2_FC) values represent increased expression in PDAC patients’ samples; NS = not significant. miRNA chosen for further validation are depicted.

We first focused our attention on miRNAs from the EVs of PDAC patients and non-affected individuals. miRNAs have garnered significant interest as a biomarker for cancer in recent years^31,32^ and have been specifically investigated for the detection and/or diagnosis of pancreatic cancer, both in PDAC tissue samples^33,34^ and EVs isolated from PDAC patients^7–12^. We were therefore interested in determining whether miRNAs contained within EVs isolated with the Vn96 synthetic peptide could also permit PDAC detection. Unsupervised hierarchal clustering analysis of the 510 miRNAs identified by our sRNAseq permitted segregation of the majority of PDAC patients from non-affected individuals (Fig. 5a), although not completely similar to clustering performed using the entire small RNA dataset (Fig. 4). 9 of 13 non-affected individuals cluster together, with the remaining 4 clustering with the majority of PDAC patients, whereas 16 of 16 PDAC patients cluster together. These data show that sRNAseq analysis of miRNA extracted from Vn96-isolated EVs permits separation of PDAC patients from non-affected individuals. By performing differential expression analysis, we identified 272 miRNA that are statistically significant (FDR < 0.05), of which 262 are upregulated with a Log_2_FC greater than + 1.0 and 7 are downregulated with a Log_2_FC less than − 1.0 (red dots, Fig. 5b).

Finally, we performed a functional analysis using the miRNA Enrichment Analysis and Annotation tool^35^. This analysis identified five statistically-significant pathways/functions that are enriched for miRNAs that we found to be differentially expressed in the EVs of PDAC patients (Table 2). Interestingly, many of these pathways/functions are known to play a role in pancreatic cancer: p53 is often mutated in PDAC^36^, perturbation of the DNA damage response and DNA replication have been characterized in a variety of cancers, including PDAC^37–39^, and pancreatic cancer stem cells rely on intact lysosomes for their survival^40^. These results further confirm that our sRNAseq analysis of small RNA extracted from Vn96-isolated EVs identifies small RNA species that play important roles in pancreatic cancer.

**Table 2:** miRNA pathway analysis.

Pathway	Number of miRNAs	p-value
p53 signalling pathway	26	0.0156
DNA damage response	16	0.0373
Lysosome	16	0.0373
DNA replication	9	0.0373
Type I diabetes mellitus	5	0.0373

We additionally performed unsupervised hierarchal clustering analyses for piRNA, tRNA, mRNA fragments, and lncRNA (fragments). Using the 659 piRNAs for clustering analysis we were able to poorly segregate the PDAC patients from non-affected individuals (Supplemental Fig. 2). Even though all non-affected individuals cluster together, only 4 of 16 PDAC patients cluster together, with the remaining 12 clustering with non-affected individuals. Unsupervised clustering of the 313 differentially-expressed tRNAs did not permit effective segregation of PDAC patients from non-affected individuals (Supplemental Fig. 3), with nearly half of each group clustering with the other group. Although not considered small RNA species, unsupervised clustering was also performed to analyze differentially-expressed mRNAs (fragments) and lncRNAs (fragments) identified by our sRNAseq analysis. At the first branch point, unsupervised clustering using mRNA (fragments) performed poorly for segregation of non-affected individuals and PDAC patients, with only 5 of 16 PDAC patients segregating from non-affected individuals, which all group together (Supplemental Fig. 4); however, unsupervised clustering using lncRNAs (fragments) proved very effective at segregating PDAC patients from non-affected individuals. In this case all non-affected individuals cluster together, whereas 14 of 16 PDAC patients cluster together, with the remaining 2 clustering with the non-affected individuals (Supplemental Fig. 5). Overall, our analyses indicate that the differential expression of some small RNA species found in plasma EVs isolated with the Vn96 synthetic peptide permits the separation of PDAC patients from non-affected individuals, albeit to varying degrees depending on the specific small RNA species analyzed.

### Differential expression of miR-21, miR-150, miR-374b, and miR-744 in Vn96-isolated EVs is associated with PDAC

We continued our investigation into differentially-expressed miRNAs found in Vn96-isolated EVs from the plasma of PDAC patients by selecting a few for follow-up validation and study. We chose to perform these experiments using droplet digital PCR (ddPCR), which allows for more accurate and reproducible measurements of the absolute copy number of miRNAs (via measurement of cDNA content) and unlike quantitative RT-PCR does not require normalization, and is thereby better suited for clinical assays^41^. The miRNAs we chose to validate were determined by three criteria: 1) highest fold change in PDAC patients versus non-affected individuals and/or 2) present in PDAC patients but absent in non-affected individuals, and/or 3) supported by the literature as a diagnostic or novel biomarker for PDAC. We therefore selected for further study miR-21, for which high expression levels in PDAC tumours have been shown to predict poor overall survival^42–44^; miR-150, whose decreased expression in PDAC tumours is associated with higher mortality^45^; miR-374b, which has been shown to contribute to cisplatin resistance in PDAC when its expression is decreased^46^; and miR-744, whose high expression in plasma has been correlated with lymph node metastasis and poor prognosis in PDAC patients^9^.

**Figure 6:**
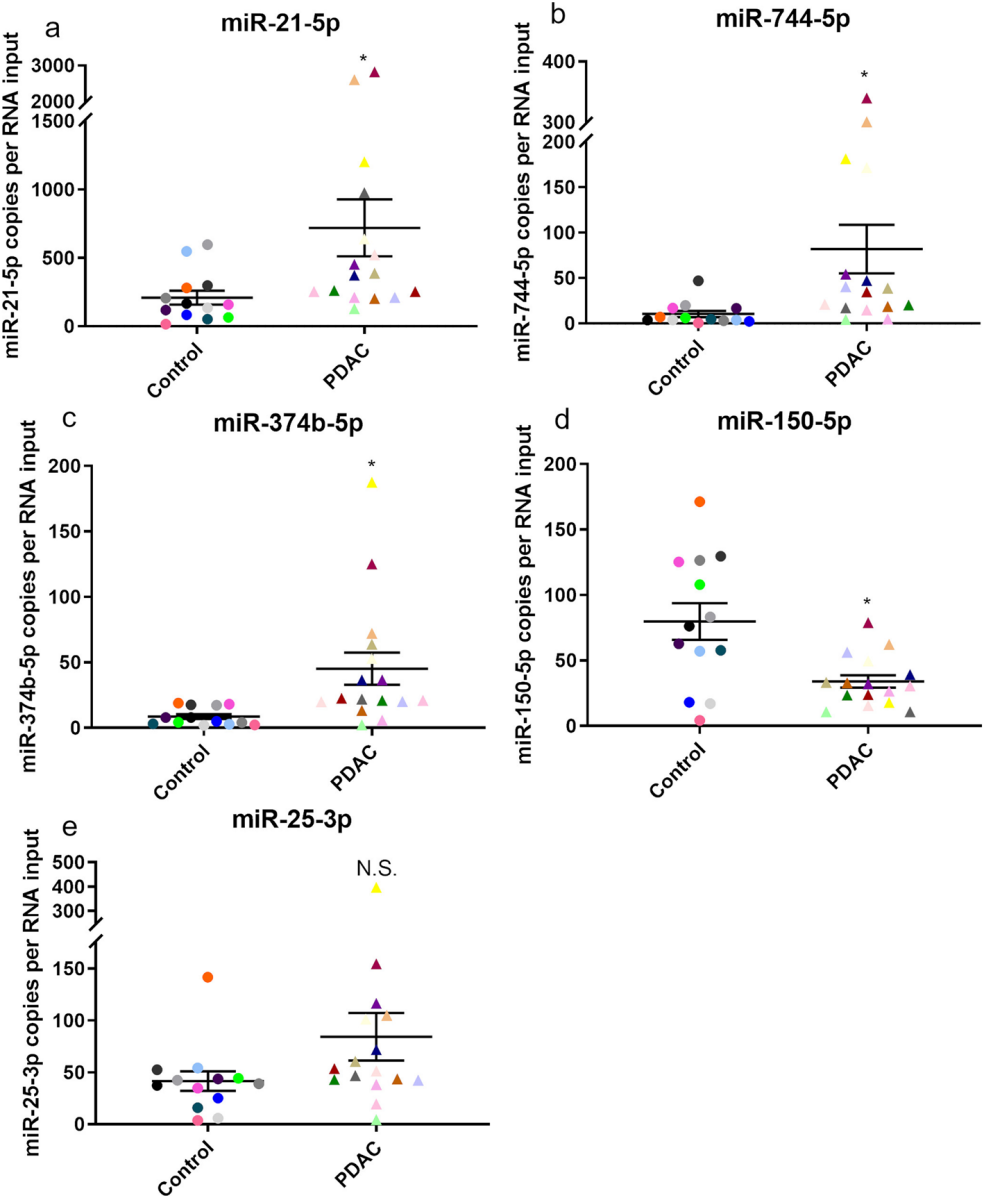
Confirmation of the differential expression of five miRNAs in Vn96-isolated EVs from non-affected individuals (Control) and PDAC patients. The concentration of the following miRNAs was measured using droplet digital PCR (ddPCR) analysis of Vn96-isolated EVs from non-affected individuals (controls; n = 13) and PDAC patients (n = 16): (**a**) miR-21-5p, (**b**) miR-744-5p, (**c**) miR-374b-5p, (**d**) miR-150-5p, and (**e**) miR-25-3p. Each coloured symbol is the average of duplicates and represents the same patient in each scatter plot. Statistical significance was calculated using a two-sample unpaired Student’s t-test (*p < 0.05, ns = not significant). Note differences in y-axis scale values.

We generated cDNA from the EV-RNA of our 13 non-affected individuals and 16 PDAC patients, which was then subjected to ddPCR analysis. Compared to non-affected individuals, all miRNAs that were found to be increased or decreased in PDAC patients’ EVs by sRNAseq were validated by ddPCR. The average miRNA copy number is significantly higher in PDAC patients for miR-21 (p = 0.0296; Fig. 6a), miR-744 (p = 0.0180; Fig. 6b), and miR-374b (p = 0.0094; Fig. 6c), whereas the average miRNA copy number is significantly lower in PDAC patients for miR-150 (p = 0.0076; Fig. 6d). The sRNAseq data showed that miR-25 expression was similar between the non-affected and PDAC patient groups, and this was also confirmed by ddPCR analysis (p = 0.1009; Fig. 6e). Together, these data confirm that miRNAs that have been previously shown to play a role in pancreatic cancer and that we identified as differentially-expressed in Vn96-isolated EVs by sRNAseq analysis do indeed show statistically-significant expression differences between the EVs of non-affected individuals and PDAC patients. We are therefore able to identify the differential expression of known miRNA biomarkers of PDAC in EVs isolated from plasma using the Vn96 synthetic peptide.

## Discussion

We set out to determine whether the differential expression of small RNA species in plasma EVs isolated with the Vn96 synthetic peptide could distinguish between pancreatic ductal adenocarcinoma (PDAC) patients and non-affected individuals. This study used derfinder for the analysis of small RNA sequencing (sRNAseq) data from EV-RNA, and our findings demonstrate the utility of this approach for analyzing EV-RNA sequencing datasets. As opposed to more common analysis approaches for sRNAseq data generated from EVs, such as exceRpt^47^, the derfinder approach allows the reporting of non-annotated chromosomal regions. In addition, derfinder does not prioritize one small RNA species over another, and thus reports regions that have overlapping annotations as multi-annotated regions. This data analysis approach allowed us to identify differentially-expressed small RNAs, regardless of RNA species, in our dataset. Indeed, our analyses of small RNA species contained within Vn96-isolated EVs permits the segregation of PDAC patients and non-affected individuals. While this segregation shows some dependency on the small RNA species analyzed, we nonetheless demonstrate that the use of a clinically-amenable EV isolation technology (Vn96) allows for the discrimination of PDAC patients from non-affected individuals. Overall, our findings align with previously-published studies on small RNA species that are associated with PDAC and provide a basis for future investigations into the utility of EV-specific small RNA species for the detection of PDAC.

Several studies have investigated the utility of EV-encapsulated biomarkers for the detection of PDAC (reviewed in^4,48,49^). Many of these studies have focused on EV-RNA as a biomarker source and have found that differential expression analysis of specific miRNAs can differentiate between PDAC and non-affected individuals. In agreement with this, we have also found that differential expression of miRNA in EVs captured with the Vn96 synthetic peptide distinguishes between PDAC and non-affected individuals (Fig. 5). Moreover, analyses of all small RNA species found within EVs (Fig. 4) and EV-lncRNA (Supplemental Fig. 5) also permit segregation of PDAC patients from non-affected individuals; however, analyses of some small RNA species, such as piRNA and tRNA, do not provide a strong segregation of PDAC patients from non-affected individuals (Supplemental Figs. 2 and 3). These findings suggest that the expression and/or packaging of specific small RNA species within EVs is highly variable in PDAC. These differences could be due to the degree to which PDAC depends on each small RNA species for its development, growth, and progression. Nonetheless, our results indicate that analysis of miRNA present within plasma EVs provides segregation of PDAC patients from non-affected individuals, with analyses of all small RNA species and lncRNA fragments in EVs having comparable segregation capability.

One challenge in applying EV-RNA biomarkers for PDAC identification, as well as other cancers, has been the diverse methods used for EV isolation in published studies, and more importantly the ability to standardize and use such methods in a clinical setting^50,51^. The variety of EV isolation methods employed has led to significant heterogeneity in the data for small RNA biomarkers of PDAC: while there is some overlap in common small RNA biomarkers identified, many have only been found in a single or a few studies^16^. This is confounded by the fact that many investigations have used ultracentrifugation to isolate EVs from patient biofluids, which is not amenable to clinical laboratory analysis because this method requires specialized equipment and a significant amount of time to complete. For these reasons we chose to employ the Vn96 synthetic peptide for EV isolation since it is a clinically-amenable technology that permits timely and reproducible EV isolation using equipment available in a diagnostic laboratory^19,22,23^. Using this approach, we identified many known miRNA markers of PDAC (Fig. 5; described in more detail below), confirming Vn96’s potential to capture EVs that contain meaningful information about PDAC status and thereby represents a step forward in applying EV-RNA biomarkers for PDAC diagnosis.

Our analyses of EV-RNA identified and validated the expression of several miRNAs that have previously been found to be associated with cancer, and PDAC specifically. For example, miR-21, which we found to be increased in the EVs of PDAC patients (Fig. 6a), is overexpressed in multiple cancers and is involved in the development of metastatic characteristics^52^. In PDAC, the overexpression of miR-21 causes increased resistance to gemcitabine, a chemotherapy administered to PDAC patients, by decreasing FasL signaling that in turn decreases apoptosis^53^. Moreover, elevated expression of exosomal miR-21 has been identified as a potential diagnostic marker for pancreatic cancer^54^, which aligns with our findings that miR-21 expression is increased in the EVs isolated from PDAC patients using our method. Both miR-744 and miR-374b, which we show are increased in PDAC patients’ EVs (Fig. 6b,c), have been identified as promising biomarkers for PDAC. miR-744 has already been identified as a diagnostic and prognostic biomarker in PDAC^9^ as well as an activator of the Wnt/β-catenin pathway^55^. Meanwhile, miR-374b affects chemoresistance to gemcitabine in PDAC by targeting mRNAs that encode anti-apoptotic proteins, such as BCL2, BIRC3 and XIAP^56^. We also observed decreased expression of miR-150 in EVs isolated from PDAC patients (Fig. 6d); decreased expression of miR-150 in PDAC tissues is associated with proliferation and metastasis, which is due to an increase in expression of the c-Myb transcription factor, as well as MUC4, which is required for cellular growth signalling and cell adhesion^45^. Furthermore, miR-150 also regulates IGF-1R and an increase in miR-150 expression negatively regulates the expression of both c-Myb and IGF-1R, leading to an increase in apoptosis^57^. We are therefore able to identify changes in the expression of miRNAs that play key roles in pancreatic cancer biology and that affect PDAC development and progression. Overall, our ability to identify the differential expression of these miRNAs in Vn96-isolated EVs from PDAC patients supports the utility of this approach for liquid biopsy-based PDAC detection via small RNA analysis.

We also found that differential-expression of lncRNA fragments contained within EVs could reliably segregate PDAC patients from non-affected individuals (Supplemental Fig. 5). lncRNAs have been the focus of intense recent study because they have been found to play a role in cancer development and progression, specifically in cancer immunity, metabolism, and metastasis, through their regulation of gene expression at the transcriptional and translational level^58^. Moreover, dysregulation of lncRNA expression has been found to be associated with both the pathology of pancreatic cancer and patient survival^59^. It is therefore not surprising that our analyses showed that differential expression of lncRNA fragments in Vn96-isolated EVs provides good segregation of PDAC patients from non-affected individuals. With this in mind, simultaneous interrogation of EV-RNA for differentially-expressed miRNAs and lncRNA fragments, as well as other small RNA species, could provide significant discriminatory power for PDAC diagnosis.

Our bioinformatic analysis identified differentially-expressed small RNAs that could separate PDAC patients from non-affected individuals (Fig. 4). Included in this dataset are small RNA sequences that are annotated as miRNA, lncRNA, tRNA and mRNA fragments, many of which have not previously been associated with PDAC, and therefore represent a set of novel PDAC biomarkers that warrant further investigation, not only for their diagnostic potential, but also for their biological roles in the development and progression of PDAC. Moreover, we also find several sequences that align with unannotated regions of the human genome (Fig. 2A), raising the possibility of unique RNA species that are differentially expressed in PDAC that may have as-yet uncharacterized roles in cancer biology. Follow-up studies should focus on validating the expression of these unannotated RNAs, as well as determining the potential role for some of these small RNAs in PDAC.

We believe our set of differentially-expressed small RNAs provides a basis for the development of a novel small RNA biomarker panel for the detection of PDAC that uses Vn96-isolated EVs from patient blood samples. Future experiments should examine whether this approach is able to discriminate between PDAC and non-affected individuals in a larger study cohort. Nonetheless, our current results indicate that analysis of small RNAs from Vn96-isolated plasma EVs is a viable method to identify PDAC patients.

## Methods

### Plasma samples

Human plasma derived from whole blood collected in EDTA-tubes was acquired from the Tumoral Biobank at the Georges-L. Dumont University Hospital Centre (Moncton, NB) and the Ontario Pancreas Cancer Study at Mount Sinai Hospital (Toronto, ON). All methods were carried out in accordance with the Canadian Research Tri-Council policy on ethical conduct for research involving humans (https://ethics.gc.ca/eng/policy-politique_tcps2-eptc2_2018.html). Approval for this study was obtained from the Research Ethics Board of the Vitalité Health Network and written informed consent was obtained from the patients. Whole blood was processed, where possible, within 30 min of collection by centrifugation at 1500×*g* for 15 min at 4 °C to separate the plasma fraction and stored at − 80 °C until use. Characteristics for all blood donors are shown in Table 1. All non-affected individuals are healthy volunteers with no known presence of any cancer.

### Vn96-mediated EV isolation and RNA extraction

Plasma was thawed at room temperature and pre-cleared at 3000×*g* for 15 min prior to EV capture. EVs were captured by peptide affinity using the Vn96 peptide (ME kit [plasma], Biosynth, US), following established protocols^19,22–24^. Briefly, pre-cleared plasma (2–4 mL) was diluted 1:1 with 1 × RNase-free phosphate buffered saline (PBS) and 150 µg of Vn96 peptide added per mL of starting plasma, then incubated with end-over-end rotation for 1 h at room temperature. Vn96-EV complexes were sedimented by centrifugation at 17,000×*g* for 15 min, then washed three times with 1 mL PBS. RNA was extracted with the miRVana miRNA Isolation kit following manufacturer’s protocol for total RNA isolation. The yield (ng per mL of starting plasma) and total RNA profile was assessed on a Fragment Analyzer System (Agilent Technologies Inc.).

### Small RNA sequencing and bioinformatic analyses

Small RNA sequencing (sRNAseq) was performed as previously described^23,26^. Briefly, small RNA libraries were prepared with 8–25 ng of RNA using the CleanTag Small RNA Library Prep kit (TriLink BioTechnologies). Raw reads from sRNAseq were processed with the Torrent Suite Software (v5.4.0), which runs the Torrent Mapping Alignment Program (TMAP). The output was converted to fastq files and the adapter sequences (TCACCGACTGCCATAGAG) were removed using TrimGalore (v 0.6.5; https://github.com/FelixKrueger/TrimGalore). Alignment was performed using STAR^60^ (version 2.7.0f.) and the GRCh38 human genome with the parameters “–outFilterScoreMinOverLread 0 –outFilterMatchNmin 16 –outFilterMatchNminOverLread 0 –outFilterMismatchNoverLmax 0.05 –alignIntronMax 1 –alignEndsType EndToEnd”. The resulting .bam files were passed through a counting method by chromosome location using the Bioconductor package derfinder^27^ (version 1.18.9). Finally, the chromosome positions with counts were annotated using multiple databases such as Gencode^61^ (v.38), pirnaDB^62^ (v.1.7.6), MINT tRNA fragment database^63^ (v.2.0), and Mirbase^64^ (v.21). R statistical environment (v3.4.1) was used for the statistical analysis of the data. Data was filtered, keeping only the chromosome locations with more than 10 reads in at least 7 non-affected individuals’ samples or 8 PDAC patients’ samples. The variance between the normalized expressed chromosome regions read counts was calculated with trimmed mean of M-values (TMM) normalization method and differential expression analysis was performed using the Bioconductor package edgeR^65^ (v3.18.1). R was used to build the following complementary figures: heatmaps were built using heatmap.2 function from ‘gplots’ with standard parameters; including the clustering (complete) and distance methods (Euclidean) with default parameters; upset plots with upset function from ‘upsetR’; MDS plots were built with plotMDS function from ‘edgeR’ library and ggplot from ‘ggplot2’ library; and the volcano plots were built using ggplot from ‘ggplot2’library. The sRNAseq data have been deposited in the NCBI Gene Expression Omnibus (GEO) under the accession number GSE221185.

### Reverse transcription and droplet digital PCR

RNA (1 ng) was reverse transcribed into cDNA using the miScript II RT kit (Qiagen) following manufacturer’s protocol. Resulting cDNA was diluted 1:8 with DNase-free water and used as a template for droplet digital PCR (ddPCR). The QX200 ddPCR system from Bio-Rad Technologies was used to measure the concentration (copies/µL) of specific miRNA as previously described^23,26^. Briefly, a volume of 5.5 µL of diluted cDNA was added to 11 µL of QX200 EvaGreen 2 × Supermix, 2.2 µL of forward primer, 1.1 µL of miScript Universal primer and 2.2 µL of nuclease-free water. Droplets were generated by mixing 20 µL reactions with 70 µL of QX200 droplet generation oil for Eva Green on a QX200 droplet generator instrument (Bio-Rad). Cycling protocol consisted of an initial 95 °C for 5 min, then 44 cycles of 95 °C for 30 s, 56 °C for 1 min and 72 °C for 2 min, then 5 min at 4 °C and 5 min at 90 °C. Ramp speed was set to 2 °C/s. Droplet fluorescence was detected on a Qx200 Droplet Reader instrument (Bio-Rad). A minimum of 10,000 droplets needed to be detected for the sample to be valid for analysis. miScript Primer Assays (Qiagen) were used for all miRNA of interest. To account for any pipetting error, samples were run in duplicate and the average copies/µL of both wells calculated. Data are expressed as the number of miRNA copies per RNA input.

### Statistical analyses

Statistical differences between conditions were evaluated using two-Sample t-test with GraphPad Prism (v9).

## Supplementary Information

Supplementary Information.

## Data Availability

The small RNA sequencing data have been deposited in the NCBI Gene Expression Omnibus (GEO) under the accession number GSE221185.
