# Supplementary material for: Small RNA sequencing analysis of peptide-affinity isolated plasma extracellular vesicles distinguishes pancreatic cancer patients from non-affected individuals
Source: Sci Rep. 2023 Jun 7;13:9251. doi: 10.1038/s41598-023-36370-3 (PMC10247738; doi:10.1038/s41598-023-36370-3)
Supplement: Supplementary file 1 — Supplementary Information. [file 41598_2023_36370_MOESM1_ESM.pdf]

SUPPLEMENTARY DATA

Title: Small RNA sequencing analysis of peptide-affinity isolated plasma extracellular vesicles distinguishes pancreatic cancer patients from non-affected individuals

Authors: Jeremy W. Roy, Gabriel Wajnberg, Alexie Ouellette, Julie Emilie Boucher, Jacynthe Lacroix, Simi Chacko, Anirban Ghosh, Rodney J. Ouellette, Stephen M. Lewis

SUPPLEMENTAL FIGURES

Supplemental Figure 1

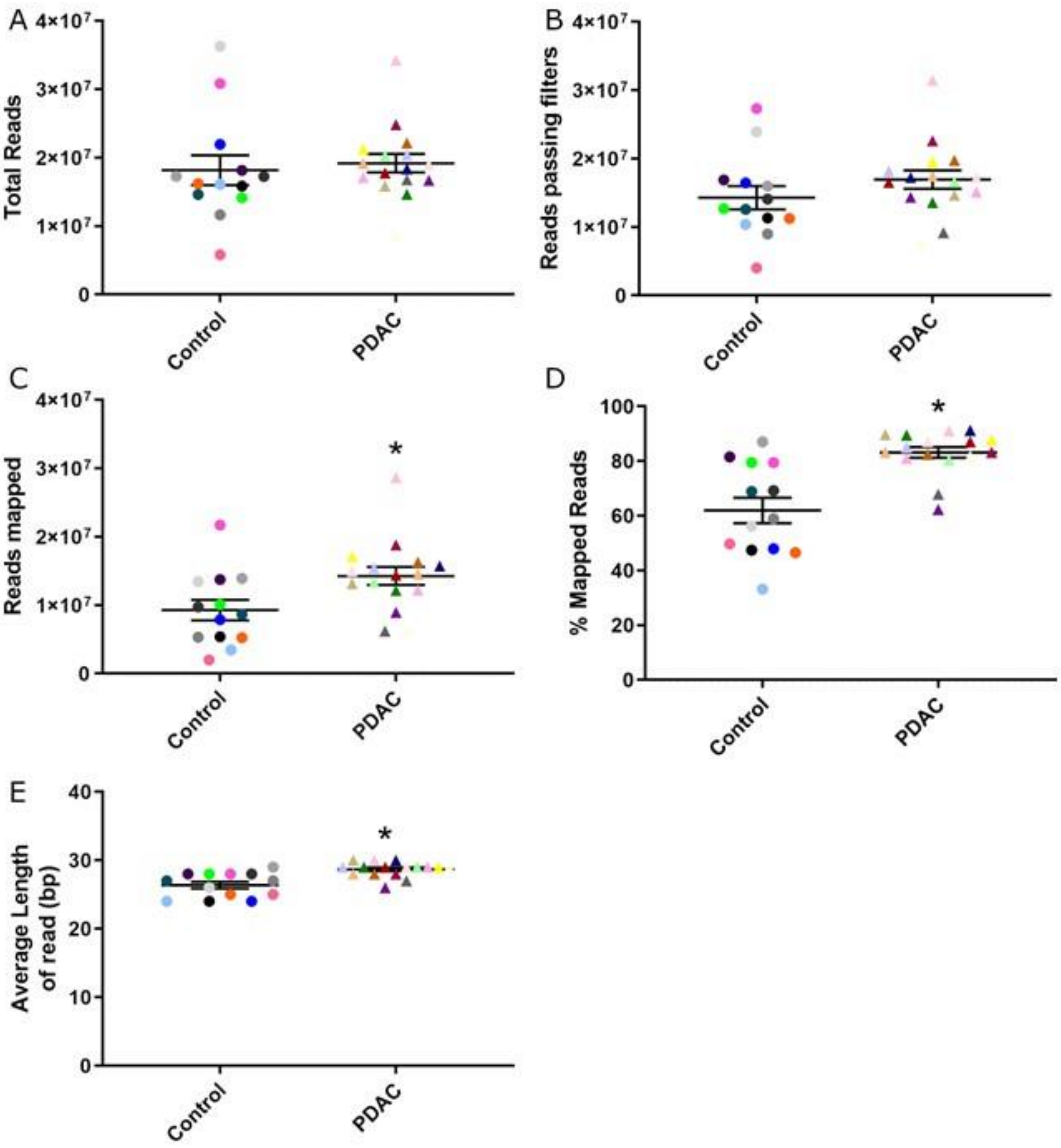

Supplemental Figure 1. Histograms depicting sRNAseq quality control characteristics. A) Total sRNA Reads, B) Reads passing filters, C) Reads mapped, D) % of mapped reads and E) average length (bp) of mapped reads in non-affected individuals and PDAC patient samples. (*$p<0.05$).

Supplemental Figure 2

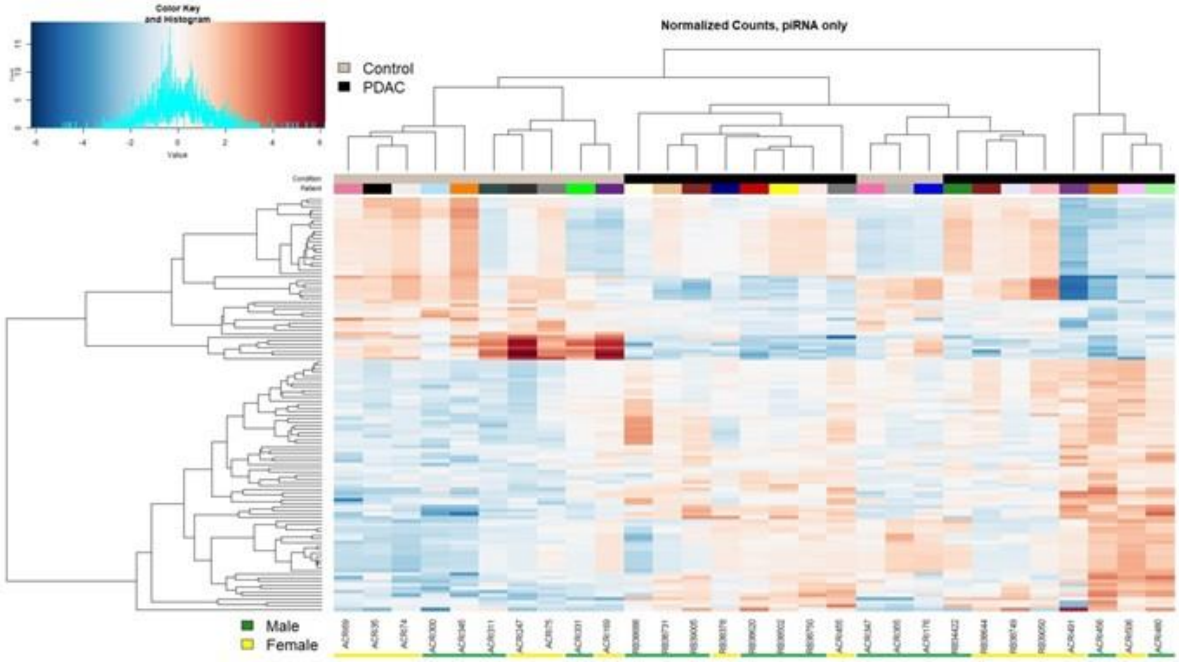

Supplemental Figure 2. Heatmap of annotated piRNA for non-affected individuals (Control; grey) and PDAC patients (black). The heatmap represents log transformed normalized (TMM) reads. Heatmap colors correspond to RNA expression as indicated in the color key: blue (down-regulated) and red (up-regulated).

Supplemental Figure 3

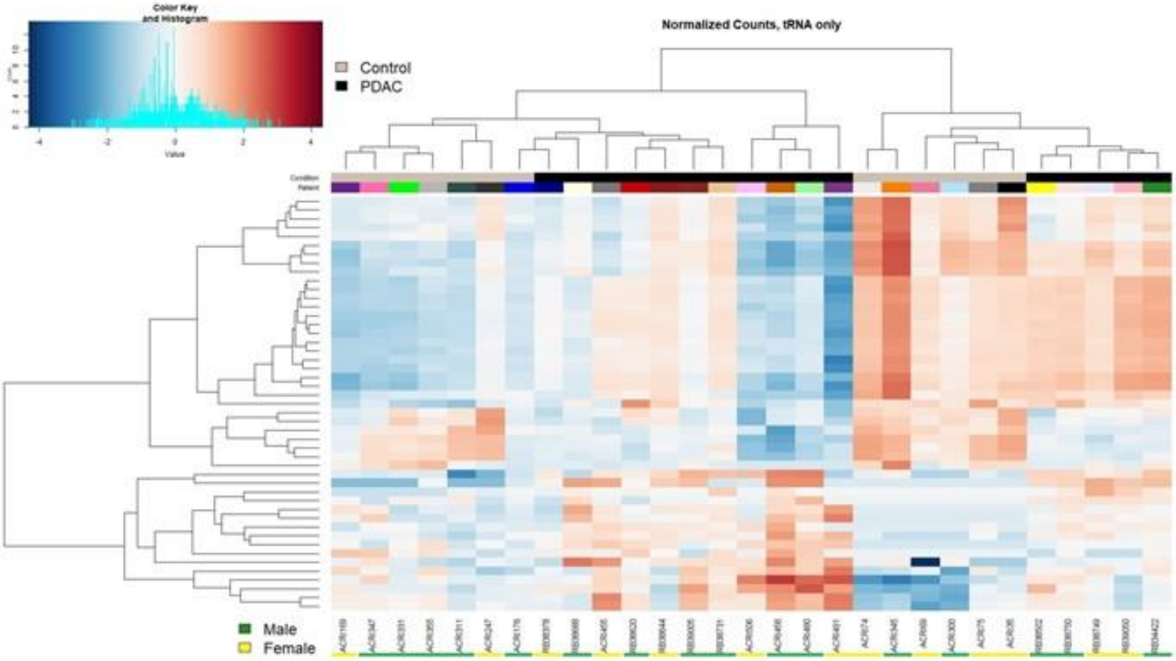

Supplemental Figure 3. Heatmap of annotated tRNA for non-affected individuals (Control; grey) and PDAC patients (black). The heatmap represents log transformed normalized (TMM) reads. Heatmap colors correspond to RNA expression as indicated in the color key: blue (down-regulated) and red (up-regulated).

Supplemental Figure 4

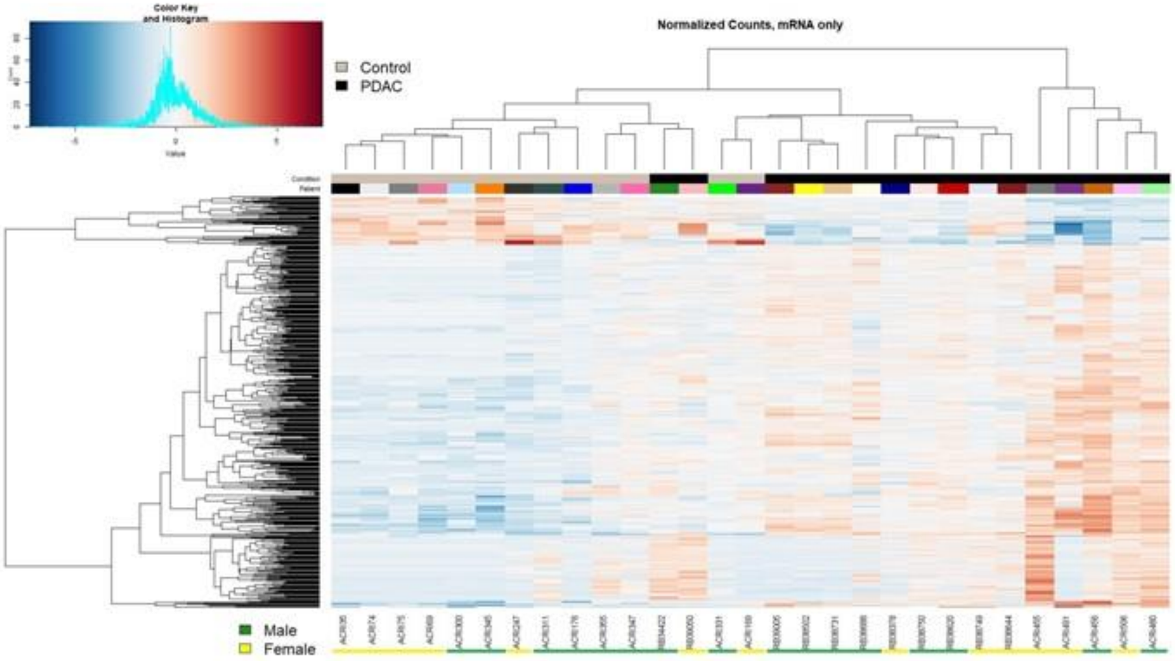

Supplemental Figure 4. Heatmap of annotated mRNA fragments for non-affected individuals (Control; grey) and PDAC patients (black). The heatmap represents log transformed normalized (TMM) reads. Heatmap colors correspond to RNA expression as indicated in the color key: blue (down-regulated) and red (up-regulated).

Supplemental Figure 5

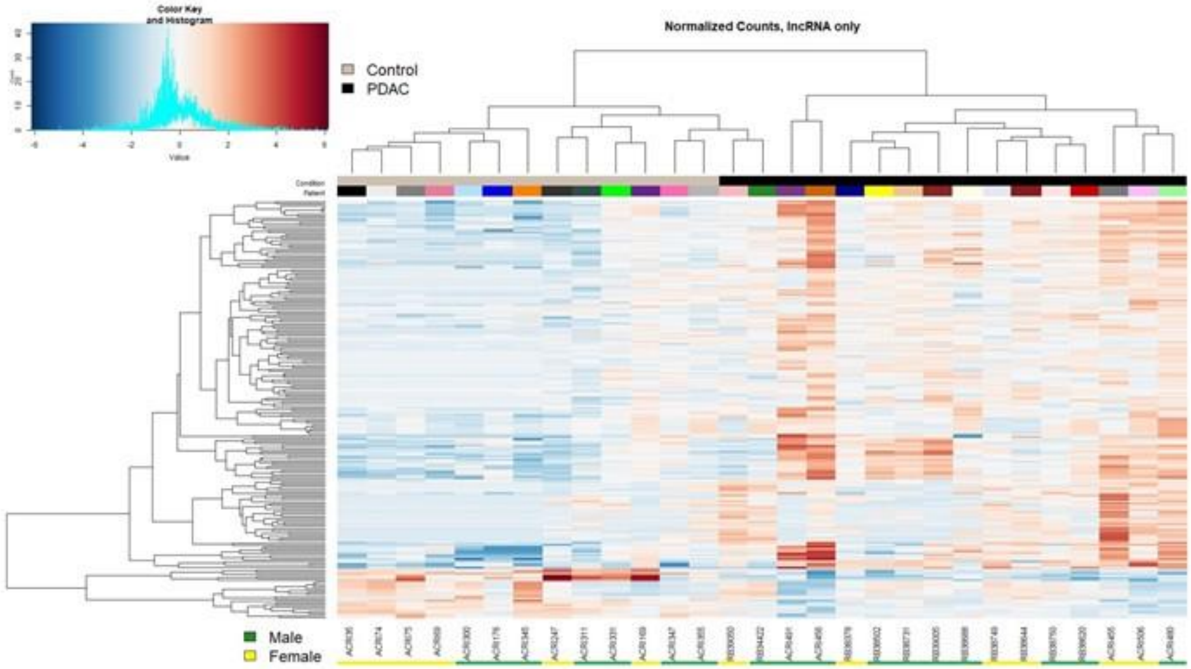

Supplemental Figure 5. Unsupervised hierarchal clustering analysis of annotated lncRNA fragments for non-affected individuals (Control; grey) and PDAC patients (black). The heatmap represents log transformed normalized (TMM) reads. Heatmap colors correspond to RNA expression as indicated in the color key: blue (down-regulated) and red (up-regulated).
